# Molecular Mechanisms of Lipopolysaccharide (LPS) Induced Inflammation in an Immortalized Ovine Luteal Endothelial Cell Line (OLENDO)

**DOI:** 10.3390/vetsci9030099

**Published:** 2022-02-24

**Authors:** Aykut Gram, Mariusz P. Kowalewski

**Affiliations:** 1 Department of Histology and Embryology, Faculty of Veterinary Medicine, Erciyes University, Kayseri 38280, Turkey; 2 Institute of Veterinary Anatomy, Vetsuisse Faculty, University of Zurich, CH-8057 Zurich, Switzerland; kowalewski@vetanat.uzh.ch; 3 Center for Clinical Studies (ZKS), Vetsuisse Faculty, University of Zurich (UZH), CH-8057 Zurich, Switzerland

**Keywords:** toll-like receptor, ovary, interleukins, PKA, PKC, MAPKs, inflammation

## Abstract

*Escherichia coli* (*E. coli*) is the most common Gram-negative bacterium causing infection of the uterus or mammary gland and is one of the major causes of infertility in livestock. In those animals affected by *E. coli* driven LPS-mediated infections, fertility problems occur in part due to disrupted follicular and luteal functionality. However, the molecular mechanisms by which LPS induces inflammation, and specifically, the role of LPS in the disruption of capillary morphogenesis and endothelial barrier function remain unclear. Here, we hypothesized that LPS may lead to alterations in luteal angiogenesis and vascular function by inducing inflammatory reactions in endothelial cells. Accordingly, OLENDO cells were treated with LPS followed by evaluation of the expression of selected representative proinflammatory cytokines: *NF-kB*, *IL6*, *IL8*, *TNFα*, and *ICAM 1*. While TNFα was not affected by treatment with LPS, transcripts of *NF-kB*, *IL6*, and *IL8* were affected in a dosage-dependent manner. Additionally, the activity of TLR2 and TLR4 was blocked, resulting in suppression of the LPS-induced expression of *ICAM 1*, *NF-kB*, *IL6*, and *IL8*. Inhibition of the PKA or MAPK/ERK pathways suppressed the LPS-stimulated expression of *NF-kB*, *IL6*, and *IL8*, whereas blocking the PKC pathway had the opposite effect. Furthermore, LPS-induced phosphorylation of Erk1 and Erk2 was inhibited when the TLR4 or MAPK/ERK pathways were blocked. Finally, LPS seems to induce inflammatory processes in OLENDO cells via TLR2 and TLR4, utilizing different signaling pathways.

## 1. Introduction

Bacterial infections of reproductive organs in dairy cows are common worldwide. Gram-negative bacteria, in particular *Escherichia coli* (*E. coli*), are the main cause of clinical metritis and mastitis and reduce reproductive performance in livestock [1,2,3]. By activating a local or systemic inflammatory response, the lipopolysaccharide (LPS) endotoxin, an outer cell wall component of Gram-negative bacteria, frequently causes infertility or subfertility in affected animals [3,4,5,6]. Moreover, luteinizing hormone (LH) secretion and pulsatility are also affected by LPS in cows and ewes [7,8,9]. LPS accumulates in ovarian follicular fluid and induces a local inflammatory response in the ovaries of animals affected by uterine or mammary gland infection [10,11,12]. As a result, inhibited follicular growth, reflected in disturbed ovarian cyclic activity, and lowered intrafollicular and circulating levels of estradiol (E2) are observed [3,4,5,6]. This is corroborated by both in vivo and in vitro observations demonstrating an LPS-dependent decrease in E2 production in bovine granulosa cells [13]. In LH-stimulated theca cells of rats, and in the bovine ovary, LPS inhibits progesterone and androstenedione synthesis [11,14]. Furthermore, administration of LPS in dairy cows reduces expression of the luteal steroidogenic acute regulatory (STAR) protein and 3β-hydroxysteroid dehydrogenase (HSD3B) [15]. Both corpus luteum (CL) size and luteal blood flow were reduced in diestric cows following the application of LPS [16]. Accordingly, the reduced luteal steroidogenic activity was associated with increased local expression of Toll-like receptor 4 (TLR4), tumor necrosis factor (TNF) α, prostaglandin F2 α synthase (PGFS/20αHSD/AKR1B1), prostaglandin E2 synthase (PTGES) and vascular endothelial growth factor (VEGFA) [17].

Pathogenic components of bacteria, such as LPS, are most commonly released upon bacterial death or degradation [18]. Toll-like receptors (TLRs) are involved in the recognition of pathogen-associated molecular patterns (PAMPs) and, thereby, the activation of innate immunity in mammalian species [19,20]. While LPS is predominantly recognized by TLR4, microbial components of Gram-positive bacteria, such as lipopeptides, peptidoglycan, and lipoteichoic acids are mostly recognized by TLR2 [19,20]. Moreover, by forming a heterodimeric complex with TLR1, TLR2 is able to distinguish different types of lipopeptides [21]. Furthermore, the LPS/TLR4-induced signaling cascade in mammalian cells induces downstream cascades, including cluster of differentiation (CD) 14 protein, myeloid differentiation factor 2 (MD2), or LPS-binding protein (LBP) [22,23]. Activation of TLRs with PAMPs stimulates nuclear translocation of (NF)-kB factor in human and bovine granulosa cells [24,25]. This is followed by the release of inflammatory mediators, TNFα and interleukins (IL) 6 and −8 [24,25,26]. LPS also induces expression of intercellular adhesion molecule-1 (ICAM 1) in bovine, human, and mouse endothelial cells [27,28,29,30].

Interestingly, bovine granulosa cells respond to LPS via both TLR4 and TLR2 [25]. Besides cattle, expression of both receptors was also detected in the CL of sheep [15,31]. In addition to luteal cells, localization of TLR4 and TLR2 was also shown in ovine and bovine endothelial cells, indicating the involvement of these cells in mediating adverse effects of LPS in the CL [15,31]. However, the impact of LPS on luteal angiogenesis and vasculogenesis in livestock has not been intensively investigated. Therefore, to gain a better understanding of the effects of infection/inflammation, and in particular of LPS, on the functionality of the CL, our laboratory recently isolated, immortalized, and characterized ovine luteal microvascular endothelial cells [31]. The new functional cell line was named OLENDO (ovine luteal endothelial cell line) [31]. With this model, we were able to show that LPS disrupts the formation of capillary-like structures and endothelial barrier function [31]. These effects appear to be associated with alterations in the gap junctional intercellular communication involving the endothelial connexin (CX) 43. Thus, LPS seems to influence the subcellular localization of CX43 in endothelial cells, resulting in the internalization of this junctional membrane protein, thereby disrupting the cell-to-cell contact [31]. Nevertheless, the molecular mechanisms by which LPS induces inflammation, and specifically, the role of LPS in disrupting the formation of capillary-like structures and endothelial barrier function remain unclear.

Here, we investigated the hypothesis that LPS leads to alterations in luteal angiogenesis and vascular function by inducing inflammatory reactions in endothelial cells.

To address this hypothesis, the inflammatory response and activation of selected, LPS-related intracellular pathways were investigated using OLENDO cells treated with LPS in the presence/absence of specific inhibitors of TLR4, TLR1/TLR2. The downstream signaling cascades were assessed by modulating the activity of specific kinases, PKA, PKC, and MAPK, in LPS treated cells. The inflammatory response was determined by checking the expression of *NF-kB*, *IL6*, *IL8*, *TNFα*, and *ICAM 1*.

## 2. Materials and Methods

### 2.1. Cell Cultures with Ovine Luteal Endothelial (OLENDO) Cells

All experiments utilized OLENDO cells, with the handling and treatment procedures based on the previously published protocol [31]. Briefly, cells were incubated in a humidified incubator at 37 °C under 5% CO_2_ and maintained in DMEM/F12 medium pH 7.2–7.4, with 10% heat-inactivated FBS, containing 100 U/mL penicillin and 20 μg/mL Endothelial Cell Growth Supplement (ECGS) (all from Chemie Brunschwig AG, Basel, Switzerland). Cells were trypsinized and transferred into 6-well plates at a concentration of 2 × 10^6^ cells per well and used for experiments 24 h later. Prior to the cell culture experiments, serum-containing medium was removed, and cells were rinsed with sterile phosphate-buffered saline (PBS) solution. A serum-free stimulation medium containing increasing concentrations of *E. coli* LPS (O55:B5; Sigma-Aldrich GmbH, Buchs, Switzerland) (1 ng/mL, 10 ng/mL, and 100 ng/mL) was then added to the wells. These dosages of *E. coli* LPS were derived from previously published reports in which mean concentrations of 176.1 ± 112 ng/mL LPS were detected in the follicular fluid of cows with clinical endometritis [4]. OLENDO cells were treated with a stimulation medium that contained specific inhibitors of PKA (H89, 25 μM), PKC (GF-109203X, 20 μM) and MAPK (UO126, 10 μM) activities (all reagents purchased from Sigma-Aldrich Chemie GmbH, St. Louis, MO, USA), alone or in combination with 100 ng/mL *E. coli* LPS. These concentrations of specific inhibitors were chosen based on previously published reports [32,33]. Selective blockers of TLR receptors from Sigma-Aldrich Chemie GmbH were used, targeted against TLR1/TLR2 and TLR4. They were applied at increasing dosages (5 μM and 10 μM for TLR1/TLR2 and 1 nM, 10 nM, and 100 nM for TLR4) in stimulation medium over 6 h and *ICAM 1* expression was assessed. This incubation period was chosen based on previous experiments showing the highest expression level of *ICAM 1* in OLENDO cells upon stimulation with 100 ng/mL LPS [31]. The lowest effective dosages of TLR4 (TAK-242, 100 nM) and TLR1/TLR2 (CU-CPT22, 5 μM) inhibitors in OLENDO cells were determined in pilot experiments, based on the lowest levels of ICAM 1 expression after 6 h of stimulation. These dosages were then used for all stimulation experiments. Non-treated cells served as controls.

### 2.2. RNA Isolation, Reverse Transcription and Qualitative RT-PCR

TRIzol^®^ reagent (Invitrogen, Carlsbad, CA, USA) was used for extraction of total RNA from OLENDO cell samples. The isolation procedures were performed according to the manufacturer’s instructions and our previously published protocols [34,35]. A NanoDrop 2000C^®^ spectrophotometer (Thermo Fisher Scientific AG, Reinach, Switzerland) was used to measure the quality and quantity of isolated total RNA. In order to remove genomic DNA contamination, DNase treatment was performed by using RQ1 RNase-free DNase (Promega, Duebendorf, Switzerland). Thereafter, RNA samples were reverse transcribed (RT) into cDNA following the manufacturer’s instructions and utilizing random hexamers as primers (Applied Biosystems by Thermo Fisher, Carlsbad, CA, USA).

Following cDNA synthesis, a conventional hot start PCR reaction was performed using the GeneAmp Gold RNA PCR Kit (Applied Biosystems by Thermo Fischer) according to the manufacturer’s protocol. The primers for *TLR1* (Table 1) were designed using Primer Express Software ver. 2.0 (Applied Biosystems by Thermo Fischer). Primers, purchased from Microsynth (Balgach, Switzerland). The PCR reaction was run with the annealing temperature set at 60 °C. Following its separation on an ethidium bromide stained 2% agarose gel, the 317 bp amplicon of *TLR1* was purified using the Qiaex II gel extraction system (Qiagen GmbH Hilden, Germany). The product was then subcloned into the pGEM-T vector (Promega) and transformed into XL1 Blue competent cells (Stratagene, La Jolla, CA, USA). Plasmid isolation and purification were performed using the Pure Yield Plasmid MidiPrep System (Promega), and isolated plasmids were then sent for commercial sequencing (Microsynth) on both strands using Sp6 and T7 promoters. Autoclaved water instead of RNA and the so-called RT minus controls were used as negative controls.

### 2.3. Semi-Quantitative RT-PCR and Data Evaluation

Semi-quantitative Real-Time (TaqMan) PCR was carried out using the ABI PRISM 7500 Sequence Detection System (Applied Biosystems) as previously described [34,35]. DNase treatment and RT were performed as described above. PCR reactions were run in duplicates in 96-well optical plates (Applied Biosystems) under the following conditions: denaturation at 95 °C for 10 min, followed by 40 cycles each of 95 °C for 15 s and 60 °C for 60 s. The sequences for forward and reverse primers and TaqMan^®^ probes labeled with 6-carboxyfluorescein (6-FAM) and 6-carboxytetramethylrhodamine (TAMRA) were designed using Primer Express Software v 2.0 (Applied Biosystems) and were bought from Microsynth. The list of self-designed primers and TaqMan^®^ probes is presented in Table 1. In order to determine the expression of *ICAM 1*, a commercially available ovine-specific TaqMan Gene Expression Assay was used, purchased from Applied Biosystems (Prod. No. Oa04658646_m1).

In order to normalize gene expression levels of the target genes, *GAPDH* and *ACTB* were used as reference genes. The sample with the lower expression level was used as a calibrator. Calculation of the relative gene expression of each target gene was performed using the comparative CT method (ΔΔCT method) according to the ABI Prism 7500 (Applied Biosystems) manufacturer’s protocol and as previously described [34,36].

### 2.4. Protein Preparation and Western Blot Analysis

Western blot analysis was performed as previously described [34,36]. Briefly, cells were washed with ice-cold PBS and harvested with NET-2 lysis buffer (50 mM Tris-HCl, PH 7.4, 300 mM NaCl, 0.05% NP-40) containing 10 μL/mL protease inhibitor cocktail (Sigma-Aldrich Chemie GmbH). Cell lysates were homogenized with a sonic distributor (Vibra-Cell, Newton, CT, USA) 75 Watt for 15 s on ice, then centrifuged at 10,000× *g* for 10 min at 4 °C to remove cell debris. The Bradford assay was used to measure the protein concentrations in the supernatants. Samples were solubilized in sample buffer (25 mmol/L Tris-Cl, pH 6.8, 1% SDS, 5% β-mercaptoethanol, 10% glycerol, 0.01% bromophenol blue) and equal amounts of proteins from the cell lysates (20–30 μg) were electrophoresed on 12% polyacrylamide gels (Bio-Rad Laboratories GmbH, Munich, Germany) at 120 V. Afterwards, proteins were blotted onto a methanol-activated polyvinylidene difluoride (PVDF) membrane (Bio-Rad) for 1 h at 100 V. The membranes were blocked with 5% low-fat dry milk in PBST (PBS/0.25% Tween-20) for 1 h at ambient temperature and incubated overnight at 4 °C with a primary antibody diluted in PBST (PBST/0.25% Tween 20) containing 2.5% skimmed milk. The primary antibodies used were rabbit polyclonal anti-human p44/42 MAPK (Erk1/2) (#9102; dilution 1:1000) and rabbit polyclonal anti-human phospho-p44/42 MAPK (Erk1/2) (#9101; dilution 1:1000), both purchased from Cell Signaling Technology, Inc., Beverly, MA, USA. The following day, after washing in PBST, membranes were incubated for 1 h with a specific horseradish peroxidase (HRP)-coupled secondary antibody at ambient temperature. Signals were developed with Immun-Star^TM^ WesternC^TM^ Chemiluminescent Kit substrate according to the manufacturer’s protocol (Bio-Rad). The detection of the signals was performed using the ChemiDoc XRS+ System (Bio-Rad) and Image Lab Software (Bio-Rad). The optical density of bands was assessed for p44/42 MAPK (Erk1/2) and phospho-p44/42 MAPK (Erk1/2), using Image Lab Software (Bio-Rad). Membranes were re-probed with an anti- ACTB antibody and the values are presented as the ratio of the optical density of the target protein relative to that of ACTB. Representative immunoblots are shown.

### 2.5. Statistical Analysis

All cell culture experiments were repeated independently at least three times, using cells from different passages.

The Shapiro–Wilk test was performed to assess the normality of the distribution of results. Since the data obtained in our current study showed a normal distribution, to assess the effects of LPS treatment on the expression of *TNFα*, *NF-kB*, *IL6*, *IL8*, and *ICAM 1*, a global comparison was performed by applying parametric one-way analysis of variance (ANOVA) with the statistical software program GraphPad 3.06 (GraphPad Software, San Diego, CA, USA). In the case of *p* < 0.05, the Tukey–Kramer multiple comparisons post-test was performed. The data are presented as the mean ± standard deviation (SD). The level of significance was considered as *p* < 0.05.

## 3. Results

### 3.1. LPS Treatment Induces Inflammatory Reaction in OLENDO Cells

**Figure 1:**
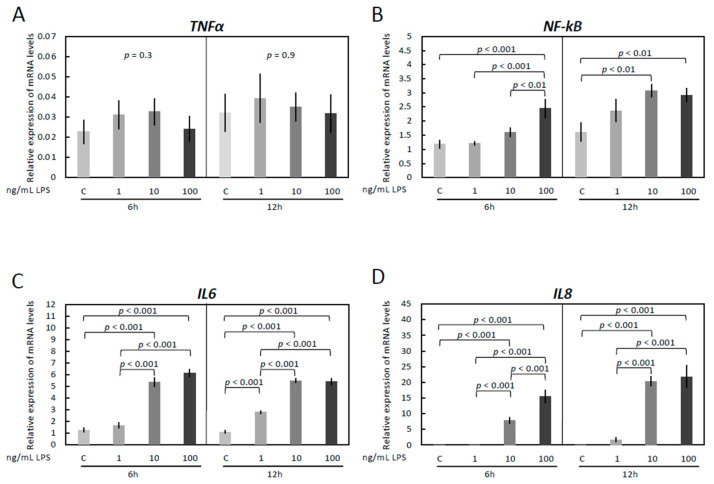
Expression of *TNFα* (**A**), *NF-kB* (**B**), *IL6* (**C**) and *IL8* (**D**) by real-time (TaqMan) PCR after treatment with 0–100 ng/mL LPS for 6 and 12 h. A parametric one-way ANOVA was applied followed by the Tukey–Kramer multiple comparisons post-test. All numerical data are presented as the means ± S.D. *p*-values < 0.05 were considered significant and are indicated.

The cells were treated with increasing concentrations of *E. coli* -derived LPS for 6 and 12 h and the gene expression of inflammation markers *TNFα*, *NF-kB*, *IL6*, and *IL8* assessed (Figure 1A–D). The respective mRNA was detectable in both treated and untreated OLENDO cells. While *TNFα* was not affected by treatment with LPS (*p* = 0.3 for 6 h and *p* = 0.9 for 12 h, Figure 1A), *NF-kB* (*p* < 0.0001 for 6 h and *p* < 0.002 for 12 h, Figure 1B), *IL6* (*p* < 0.0001 for both 6 h and 12 h, Figure 1C) and *IL8* (*p* < 0.0001 for both 6 h and 12 h, Figure 1D), were affected in a dosage-dependent manner. In greater detail (Figure 1B), although 100 ng/mL LPS significantly stimulated *NF-kB* mRNA expression over a 6 h time course (*p* < 0.001), a lower dosage of LPS (10 ng/mL, *p* < 0.01) was effective in a 12 h stimulation experiment. The expression of *IL6* (Figure 1C) increased significantly over controls within 6 h, in response to both the lower (10 ng/mL) and higher (100 ng/mL) LPS concentrations (*p* < 0.001 for both). This effect was also apparent in cells incubated for 12 h, with the respective mRNA levels increasing gradually in response to all LPS dosages (*p* < 0.001 for 1, 10 and 100 ng/mL LPS), compared with the respective control. Similarly, mRNA levels of *IL8* resembled that of *IL6* and were significantly upregulated at 6 and 12 h in response to 10 ng/mL and 100 ng/mL LPS (*p* < 0.001 for both dosages) compared with the respective controls (Figure 1D).

### 3.2. OLENDO Cells Respond to E. coli LPS through TLR1/TL2 and TLR4 Pathways

**Figure 2:**
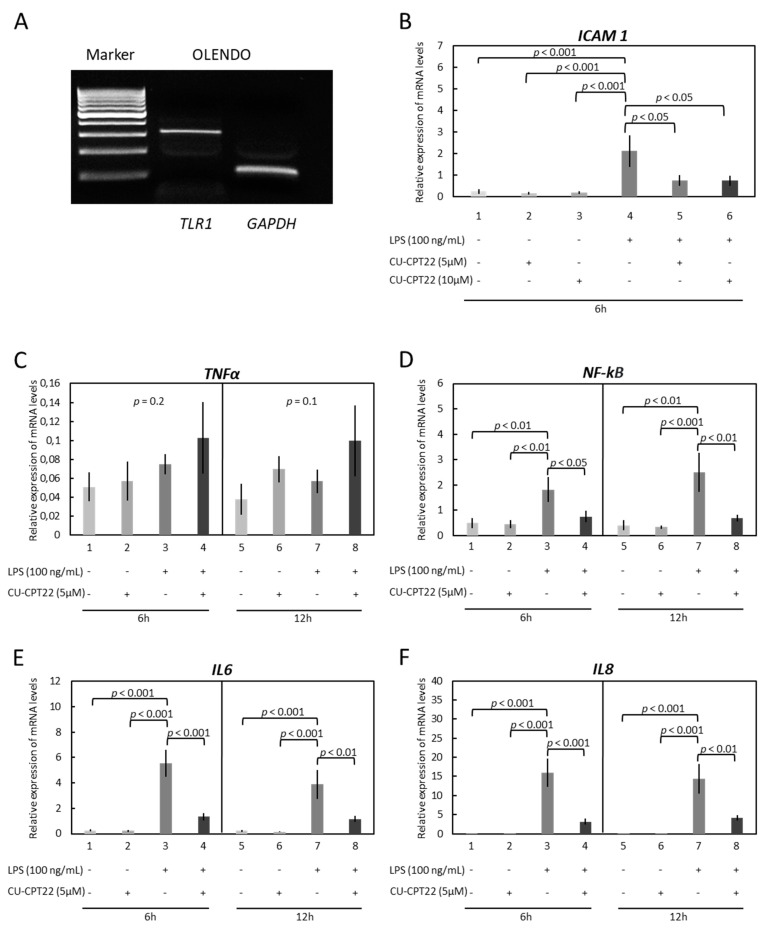
The expression of *TLR1* and effects of LPS treatment and functional blocking of TLR1/TLR2 on expression of *ICAM 1*, *TNFα*, *NF-kB*, *IL6*, and *IL8* in immortalized OLENDO cells. (**A**) The expression of *TLR1* in OLENDO cells (317 bp) by qualitative PCR (M = marker, *GAPDH* as internal control). Expression of *ICAM 1* (**B**), *TNFα* (**C**), *NF-kB* (**D**), *IL6* (**E**), and *IL8* (**F**) as determined by real-time (TaqMan) PCR. A selective blocker of TLR1/TLR2 (CU-CPT22) was used as described in the Materials and Methods. A parametric one-way ANOVA was applied followed by the Tukey–Kramer multiple comparisons post-test. All numerical data are presented as the means ± S.D. *p*-values < 0.05 were considered significant and are indicated. The original western blotting figures in Figure 2A can be found in Figure S1.

**Figure 3:**
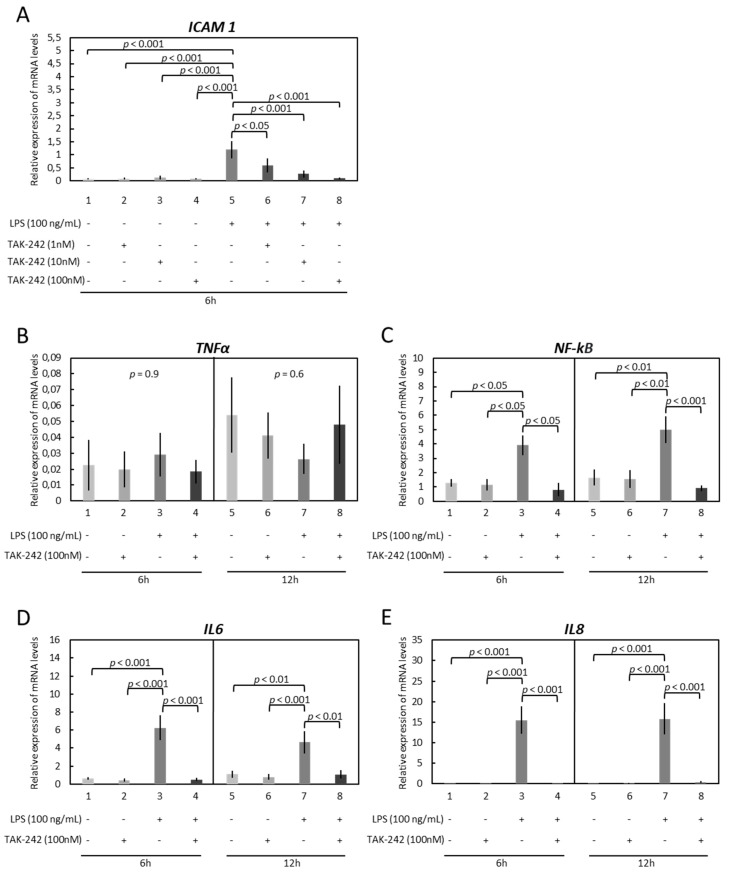
Effects of LPS treatment and functional blocking of TLR4 on expression of *ICAM 1*, *TNFα*, *NF-kB*, *IL6*, and *IL8* in immortalized OLENDO cells. Expression of *ICAM 1* (**A**), *TNFα* (**B**), *NF-kB* (**C**), *IL6* (**D**), and *IL8* (**E**) as determined by real-time (TaqMan) PCR. A selective blocker of TLR4 (TAK-242) was used as described in the Materials and Methods. A parametric one-way ANOVA was applied followed by the Tukey–Kramer multiple comparisons post-test. All numerical data are presented as the means ± S.D. *p*-values < 0.05 were considered significant and are indicated.

The potential involvement of TLRs in the LPS-induced inflammatory reaction in OLENDO cells was investigated by applying commercially available blockers of TLR1/-2 and TLR4 (Figure 2 and Figure 3). While the presence of TLR2 and TLR4 in OLENDO cells has been shown previously [31], here the basal availability of TLR1 in these cells was confirmed by conventional PCR (Figure 2A), and its expression was stably maintained throughout passages. Next, cells were treated with LPS following the application of the specific functional blockers of TLR1/TLR2 (CU-CPT22) or TLR4 (TAK-242), and the gene expression of proinflammatory cytokines *ICAM 1*, *TNFα*, *NF-kB*, *IL6*, and *IL8* was assessed.

Apart from *TNFα*, the expression of all factors was significantly upregulated in response to LPS treatment. Similarly, both blockers significantly suppressed LPS-driven gene expression. In detail, whereas LPS induced the expression of *ICAM 1* (*p* < 0.001), the TLR1/TLR2 blocker (CU-CPT22) significantly reduced its levels for both dosages tested (Figure 2B).

Similarly, the LPS-induced gene expression levels of *NF-kB* (*p* < 0.05 for 6 h and *p* < 0.01 for 12 h, respectively), *IL6* (*p* < 0.001 for 6 h and *p* < 0.01 for 12 h, respectively), and *IL8* (*p* < 0.001 for 6 h and *p* < 0.01 for 12 h, respectively), were significantly suppressed in response to CU-CPT22 (Figure 2D–F). 

Expression patterns resembling those observed in response to the TLR1/-2 blocker were observed in experiments utilizing the TLR4 blocker (TAK-242). Thus, TAK-242 significantly suppressed the LPS-stimulated expression of *ICAM 1*, *NF-kB*, *IL6*, and *IL8* (Figure 3A–E). In greater detail, the ICAM 1 levels decreased gradually, in a dosage-dependent manner, in response to 1 nM (*p* < 0.05), 10 nM (*p* < 0.001), and 100 nM (*p* < 0.001) of the blocker (Figure 3A). The following results were observed for the other genes: *NF-kB* (*p* < 0.05 for 6 h and *p* < 0.001 for 12 h), *IL6* (*p* < 0.001 for 6 h and *p* < 0.01 for 12 h), and *IL8* (*p* < 0.001 for 6 h and *p* < 0.001 for 12 h) (Figure 3C–E).

### 3.3. Modulation of Functionality of Kinases (PKA, PKC, MAPKs) Alters the Expression of Inflammatory Markers in OLENDO Cells

**Figure 4:**
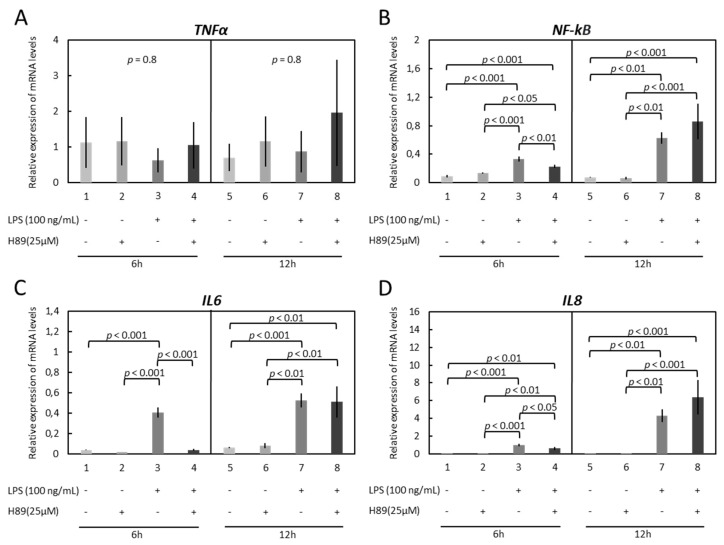
Effects of LPS treatment and inhibition of PKA (H89) activity on expression of *TNFα*, *NF-kB*, *IL6* and *IL8* in immortalized OLENDO cells. Expression of *TNFα* (**A**), *NF-kB* (**B**), *IL6* (**C**), and *IL8* (**D**) as determined by real-time (TaqMan) PCR. Inhibition of PKA (H89) activity was performed as described in the Materials and Methods. A parametric one-way ANOVA was applied followed by the Tukey–Kramer multiple comparisons post-test. All numerical data are presented as the means ± S.D. *p*-values < 0.05 were considered significant and are indicated.

**Figure 5:**
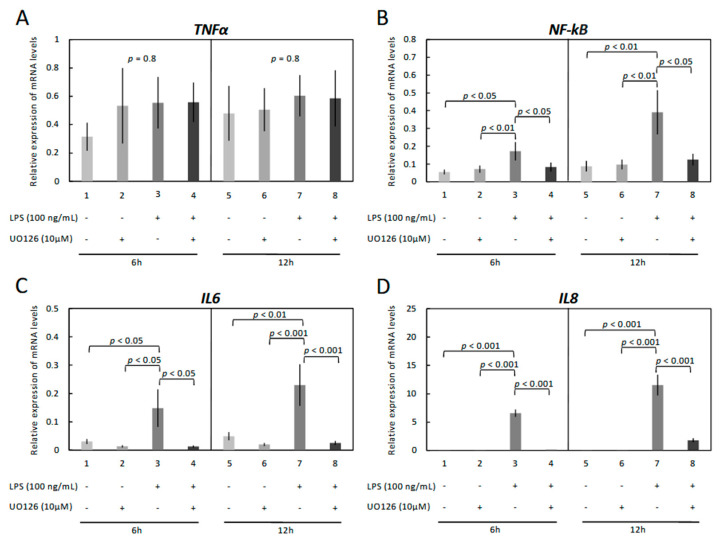
Effects of LPS treatment and inhibition of MAPK (UO126) activity on expression of *TNFα*, *NF-kB*, *IL6* and *IL8* in immortalized OLENDO cells. Expression of *TNFα* (**A**), *NF-kB* (**B**), *IL6* (**C**), and *IL8* (**D**) as determined by real-time (TaqMan) PCR. Inhibition of MAPK (UO126) activity was performed as described in the Materials and Methods. A parametric one-way ANOVA was applied followed by the Tukey–Kramer multiple comparisons post-test. All numerical data are presented as the means ± S.D. *p*-values < 0.05 were considered significant and are indicated.

**Figure 6:**
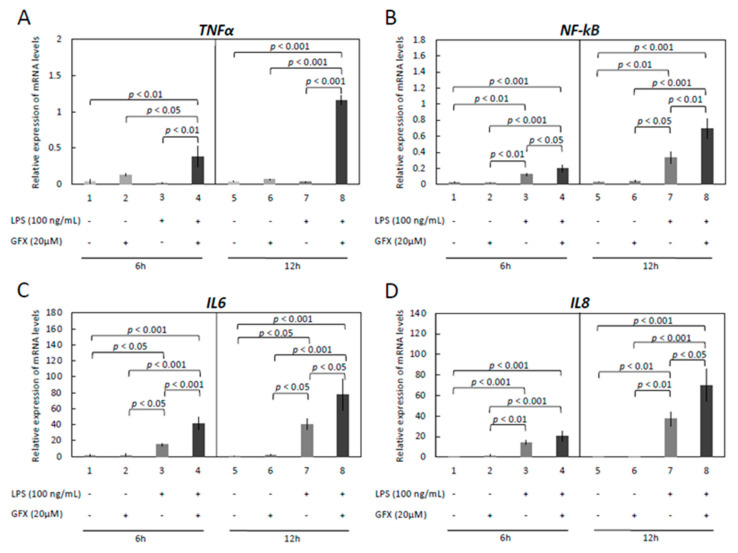
Effects of LPS treatment and inhibition of PKC (GF-109203X) activity on expression of *TNFα*, *NF-kB*, *IL6* and *IL8* in immortalized OLENDO cells. Expression of *TNFα* (**A**), *NF-kB* (**B**), *IL6* (**C**), and *IL8* (**D**) as determined by real-time (TaqMan) PCR. Inhibition of PKC (GF-109203X; GFX) activity was performed as described in the Materials and Methods. A parametric one-way ANOVA was applied followed by the Tukey–Kramer multiple comparisons post-test. All numerical data are presented as the means ± S.D. *p*-values < 0.05 were considered significant and are indicated.

The downstream signaling cascades in LPS treated OLENDO cells were assessed by modulating the activity of the kinases, PKA, PKC, and MAPK, via specific blockers, H89, UO126 and GF-109203X, respectively (Figure 4, Figure 5 and Figure 6). The inflammatory response was evaluated by checking the gene expression levels of selected proinflammatory cytokines: *TNFα*, *NF-kB*, *IL6*, and *IL8*.

While *TNFα* was not affected by treatment with H89 (*p* = 0.8 for both 6 h and 12 h, Figure 4A), inhibition of the PKA pathway suppressed the LPS-stimulated expression of *NF-kB* (*p* < 0.01), *IL6* (*p* < 0.001) and *IL8* (*p* < 0.05) in a 6 h time course (Figure 4B–D). However, this effect of H89 was not apparent in cells incubated for 12 h (Figure 4B–D).

Similarly, blocking the MAPK/ERK pathway significantly suppressed the LPS-stimulated gene expression levels of *NF-kB*, *IL6* and *IL8.* In greater detail, LPS induced mRNA expression of *NF-kB* (*p* < 0.05 for both 6 h and 12 h, respectively), *IL6* (*p* < 0.05 for 6 h and *p* < 0.001 for 12 h, respectively), and *IL8* (*p* < 0.001 for both 6 h and 12 h), but their expression was significantly decreased in response to UO126 (Figure 5B–D).

In contrast to the effects observed in response to H89 and UO126 treatment, blocking of the PKC pathway significantly potentiated the LPS-stimulated gene expression levels of *NF-kB*, *IL6* and *IL8*, as well as the expression of *TNFα* (Figure 6A–D). In detail, the LPS-induced gene expression levels of *NF-kB* (*p* < 0.05 for 6 h and *p* < 0.01 for 12 h, respectively) and *IL6* (*p* < 0.001 for 6 h and *p* < 0.05 for 12 h, respectively) were significantly potentiated in response to GFX. However, this effect of GFX-109203X was apparent for *IL8* only in cells incubated for 12 h, with the respective mRNA levels significantly upregulated in response to the dosage used (*p* < 0.05) (Figure 6D).

### 3.4. LPS Induces Phosphorylation of p44/42 MAPK (Erk1/2) in OLENDO Cells

**Figure 7:**
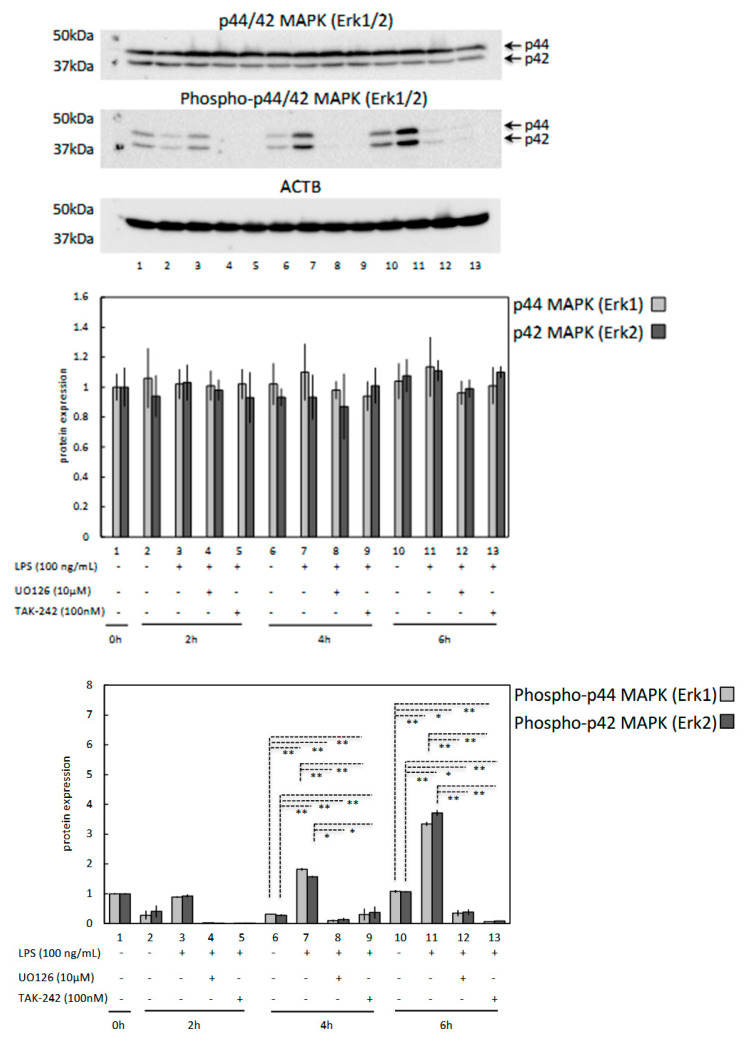
Effects of LPS treatment and MAPK (UO126, 10 μM) and TLR4 (TAK-242, 100 nM) inhibitors on protein expression of p44/42 MAPK (Erk1/2) and phosphorylated p44/42 MAPK (Erk1/2) proteins for 0–6 h. ACTB expression (45 kDa) was used as a loading control. Representative immunoblots are shown. Lower panels represent densitometric values (standardized optical density; SOD) for Erk1 (p44), Erk2 (p42) and pErk1 (p44), pErk2 (p42) expression normalized against ACTB. A parametric one-way ANOVA was applied followed by the Tukey–Kramer multiple comparisons post-test. All numerical data are presented as the means ± S.D. *p*-values < 0.05 were considered significant: * indicates *p* < 0.01, ** indicates *p* < 0.001. The original western blotting figures in Figure 7 can be found in Figure S2.

To examine the possible effect of LPS on MAPK (Erk1/2) activation in OLENDO cells, 100 ng/mL LPS was applied to the cells, and cell extracts collected at different time points were processed for western blotting to detect the expression and activation (i.e., phosphorylation) of MAPK (Erk1/2). As shown in Figure 7, activation of MAPK (pErk1/2) was seen 4 h after LPS treatment and remained stable for up to 6 h. However, this effect of LPS was attenuated for MAPK (pErk1/2) in the presence of 10 μM MAP kinase inhibitor (UO126) or 100 nM TLR4 blocker (TAK-242) in the stimulation medium. Interestingly, neither of the blockers nor LPS had a significant effect on the expression of total (unphosphorylated) MAPK (Erk1/2) (Figure 7). 

## 4. Discussion

Bacterial infections of the uterus or mammary gland may affect the structure and function of the CL by involving the functionality of the vascular bed. Although the expression of TLR2 and TLR4 have been found in luteal endothelial cells of different mammalian species, the impact of LPS on luteal angiogenesis and vasculogenesis in livestock is not well understood. Therefore, by using the previously established OLENDO cell line [31], this study was designed to examine the effects of LPS on the endothelial cell-mediated immune response. Building on the previous study that proved the presence of TLR2 and TLR4 in OLENDO cells, here the expression of TLR1 was also confirmed in OLENDO cells and remained stable throughout the passages. The expression of the three receptors in OLENDO cells suggests the involvement of luteal endothelial cells in recognition of PAMPs associated with Gram-negative and Gram-positive bacteria. While TLR4 is activated by LPS, TLR2, by forming a heterodimeric complex with TLR1, is involved in the recognition of microbial components of Gram-positive bacteria, such as lipopeptides, peptidoglycan, and lipoteichoic acids [19,20,21]. An LPS-induced inflammatory reaction via TLR4 has been demonstrated in human umbilical vein endothelial cells, lung microvascular endothelial cells, and coronary artery endothelial cells [37,38,39]. In line with this, treatment of OLENDO cells with LPS significantly increased expression of *NF-kB*, *IL6*, and *IL8* mRNA in a dose-dependent manner within 6 h of stimulation. These effects were maintained in 12 h stimulation experiments, with increased response towards lower concentrations of LPS, implicating time-dependent effects. However, TNFα was not affected by treatment with LPS in OLENDO cells. Effects of LPS treatment on the expression of TNFα may vary depending on particular cell types. For example, in human umbilical vein endothelial cells (HUVEC) [40] and human adipocytes [41], in contrast to OLENDO cells and human airway leucocytes [42], LPS induced expression of TNFα. The regulatory mechanisms behind the differential action of LPS on expression of TNFα in different cell types remain unclear, but certainly deserve further investigations. Furthermore, in the present study, we were able to show that LPS-induced elevation of *NF-kB* and cytokines *IL6* and *IL8* is modulated by both TLR2 and TLR4 receptors. Accordingly, the suppression of TLR2 and TLR4 activity resulted in a significant reduction of their expression.

Additionally, in the present study with OLENDO cells, the effects of TLR1/2 and TLR4 receptors on LPS-induced *ICAM 1* expression were assessed. ICAM 1 is an important adhesion molecule involved in the attachment and recruitment of leukocytes to the endothelium [30,43,44]. Its activation induces inflammation and vascular disruption, as shown e.g., in rat brain endothelial cells or human umbilical vein endothelial cells [30,43,44]. Similarly, recent results from our group showed that LPS disrupts in vitro capillary morphogenesis and endothelial barrier function, in association with increased *ICAM 1* expression and altered gap junctional intercellular communication, mediated particularly by Cx43 in OLENDO cells [31]. Interestingly, as presented here, blocking of TLR1/2 or TLR4 receptors significantly decreased the expression of *ICAM 1* in LPS-treated OLENDO cells in a dose-dependent manner. This strongly suggests the involvement of these receptors in LPS induced inflammatory processes and disruption of capillary morphogenesis and endothelial barrier function in luteal endothelial cells.

Moreover, MAPK/ERK regulated pathways are known to play important roles in the regulation of inflammation-associated cytokine and chemokine production, as shown e.g., in mouse splenocytes, human mesangial and proximal tubular cells and peripheral mononuclear cells [45,46,47]. For instance, activation of TLRs with PAMPs in bovine and human granulosa cells stimulates downstream cascades involving activation of mitogen-activated protein kinases (MAPK) and nuclear translocation of NF-kB [24,25]. In the present study, we were able to show that the functional suppression of the MAPK/ERK pathway diminishes the LPS-induced expression of proinflammatory cytokines in OLENDO cells. This is also consistent with observations made in other studies, in which exposure of immune cells to PAMPs activated the MAPK/ERK pathways and, thereby, production of inflammatory cytokines and chemokines [48]. Moreover, our data demonstrate that LPS induced activation of Erk1/2 phosphorylation was diminished by a MAPK/ERK pathways inhibitor (UO126) without affecting protein expression of ERK1/2 in OLENDO cells. Since, LPS induces phosphorylation of ERK 1/2 and production of pro-inflammatory cytokines such as *IL6*, and chemokines such as *IL8* in OLENDO cells, this strongly implicates the importance of the MAPK/ERK signaling in LPS associated inflammatory effects in luteal endothelial cells. Additionally, in the present study, we were able to demonstrate that the LPS-driven phosphorylation of Erk1/2 is modulated by TLR4. The functional suppression of its activity diminished the LPS-induced phosphorylation of Erk1/2 in OLENDO cells. Therefore, the present study further emphasizes the previously shown interaction between TLR4 and MAPK/ERK pathways in different cell types, e.g., in human monocytes or bovine granulosa cells [10,49]. However, the possible involvement of TLR2 in LPS-mediated activation of MAPK/ERK pathways in OLENDO cells and other cell types also appears interesting and requires further investigation.

Although the importance of the MAPK/ERK cascade in LPS-mediated inflammation was investigated intensively in different cell types, the potential role of other kinases in this process remains to be elucidated. Some information derives from studies with human gingival fibroblast cells, mouse pituitary cells or human bladder epithelial cells and monocytes, in which blocking of the PKA pathway resulted in a decrease in LPS-induced production of cytokines such as IL6, and TNFα [50,51,52,53]. Here, by making use of OLENDO cells, we assessed the potential role of the PKA and PKC pathways in LPS-mediated inflammation in luteal endothelial cells. Similar to the situation observed in human and mouse models [50,51,52,53], we found a significant reduction of the LPS response in OLENDO cells following treatment with the PKA blocker H89. With this, we have shown for the first time the functional involvement of the PKA pathway in regulating LPS induced inflammation in luteal endothelial cells. Another protein kinase family member, PKC, was also implicated in regulating the LPS- and other TLR ligand-induced inflammatory processes in human monocytes, mouse macrophages, dendritic cells, and neutrophils [54,55,56,57]. The blocking of its function induces the production of inflammatory mediators such as TNFα and IL1B in human monocytes [56]. Accordingly, in our experiments, cells were treated with LPS alone or in combination with the PKC blocker and the expression of proinflammatory cytokines was assessed. Notably, the PKC blocker appeared to amplify the gene expression of LPS-induced proinflammatory cytokines. While these findings suggest a modulatory role of PKC in the LPS-inflammatory response in endothelial cells towards increasing inflammation, seemingly contrary to those effects mediated via PKA, it needs to be emphasized that our in vitro observations need further confirmation in vivo. 

## 5. Conclusions

In conclusion, luteal endothelial cells, represented in our study by OLENDO cells, appear to be involved in the recognition of PAMPs associated with Gram-negative and Gram-positive bacteria in the CL through the expression of TLR1, TLR2 and TLR4. In particular, the TLR2 and TLR4 systems, by mediating the production of pro-inflammatory cytokines, appear to be important players mediating the adverse effects of LPS in CL. The underlying molecular mechanisms, interactions and communication between different signaling cascades need further study as they may reveal important mechanisms underlying the regulation of LPS-induced inflammation, resulting e.g., in disruption of luteal functionality and impaired ovarian cyclic activity in domestic mammalian species and thereby bearing great clinical relevance.

## Figures and Tables

**Table 1:** List of primers used for the conventional and semi-quantitative real time (TaqMan) RT-PCR.

Gene	Primer Sequence	Product Length (bp)	Accession Number
*TLR1*	Forward: 5′-GCC ACC CTA CTC TGA ACC TC-3′ Reverse: 5′-ACT CAC TGT GGT GCT GAC TG-3′	317	NM_001135060.2
*TNFα*	Forward: 5′-GCC CTG GTA CGA ACC CAT CT-3′ Reverse: 5′-CAG GTA TTC CGG CAG GTT GA-3′ TaqMan probe: 5′-CCA GCT GGA GAA GGG AGA TCG CCT C-3′	91	NM_001024860.1
*NF-kB*	Forward: 5′-CAT CGA GGT TCG GTT CTA CGA-3′ Reverse: 5′-GGA GGT GTC CGG AAC ACA AT-3′ TaqMan probe: 5′-TGA GAA TGG ATG GCA AGC CTT TGG G-3′	108	AF283892.1
*IL6*	Forward: 5′-CCA CTG CTG GTC TTC TGG AGT ATC-3′ Reverse: 5′-CTC TGC AAC TCC ATG ACA GTT TCC-3′ TaqMan probe: 5′-ACC TGG ACT TCC TCC AGA ACG AGT TTG AG-3′	91	NM_001009392.1
*IL8*	Forward: 5′-CTC TGT GTG AAG CTG CAG TTC TG-3′ Reverse: 5′-GCA GTG TGG CCC ACT CTC AA-3′ TaqMan probe: 5′-ACA CAT TCC ACA CCT TTC CAC CCC A-3′	131	NM_001009401.2
*GAPDH*	Forward: 5′-GGC ACA GTC AAG GCA GAG AAC-3′ Reverse: 5′-CAC GTA CTC AGC ACC AGC ATC A-3′ TaqMan probe: 5′-AAG GCC ATC ACC ATC TTC CAG GAG C-3′	114	NM_001190390.1
*ACTB*	Forward: 5′-AGA GGC ATC CTG ACC CTC AA-3′ Reverse: 5′-GTT GTA GAA GGT GTG GTG CCA GAT-3′ TaqMan probe: 5′-TAC CCC ATT GAG CAC GGC ATT GTC A -3′	93	U39357.1

## Data Availability

The data presented in this study are available on request from the corresponding author.

## Supplementary Materials

The following are available online at https://www.mdpi.com/article/10.3390/vetsci9030099/s1. Figure S1: Original unedited image indicating the expression of TLR1 in OLENDO cells (317 bp) by qualitative PCR (M = marker, GAPDH as internal control) used in Figure 2A of the manuscript. Figure S2: Original unedited images indicating p44/42 MAPK (Erk1/2), phosphorylated p44/42 MAPK (Erk1/2) and ACTB for representative western blots used in Figure 7 of the manuscript.
