# Supplementary figures and images for: Molecular Mechanisms of Lipopolysaccharide (LPS) Induced Inflammation in an Immortalized Ovine Luteal Endothelial Cell Line (OLENDO)
Source: Vet Sci. 2022 Feb 24;9(3):99. doi: 10.3390/vetsci9030099 (PMC8950530; doi:10.3390/vetsci9030099)

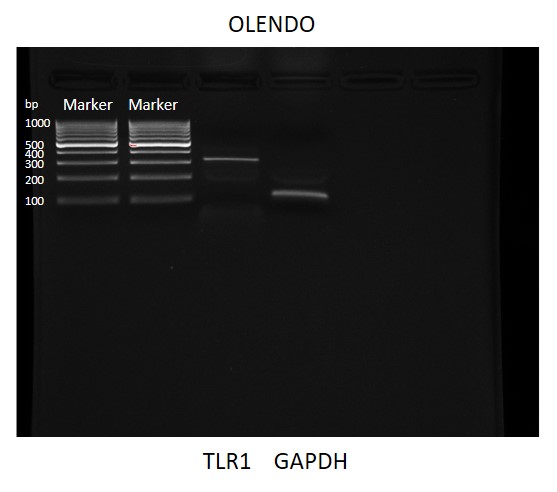

Supplement: Supplementary file 1 [file vetsci-09-00099-s001.zip › Figure S1.jpg]

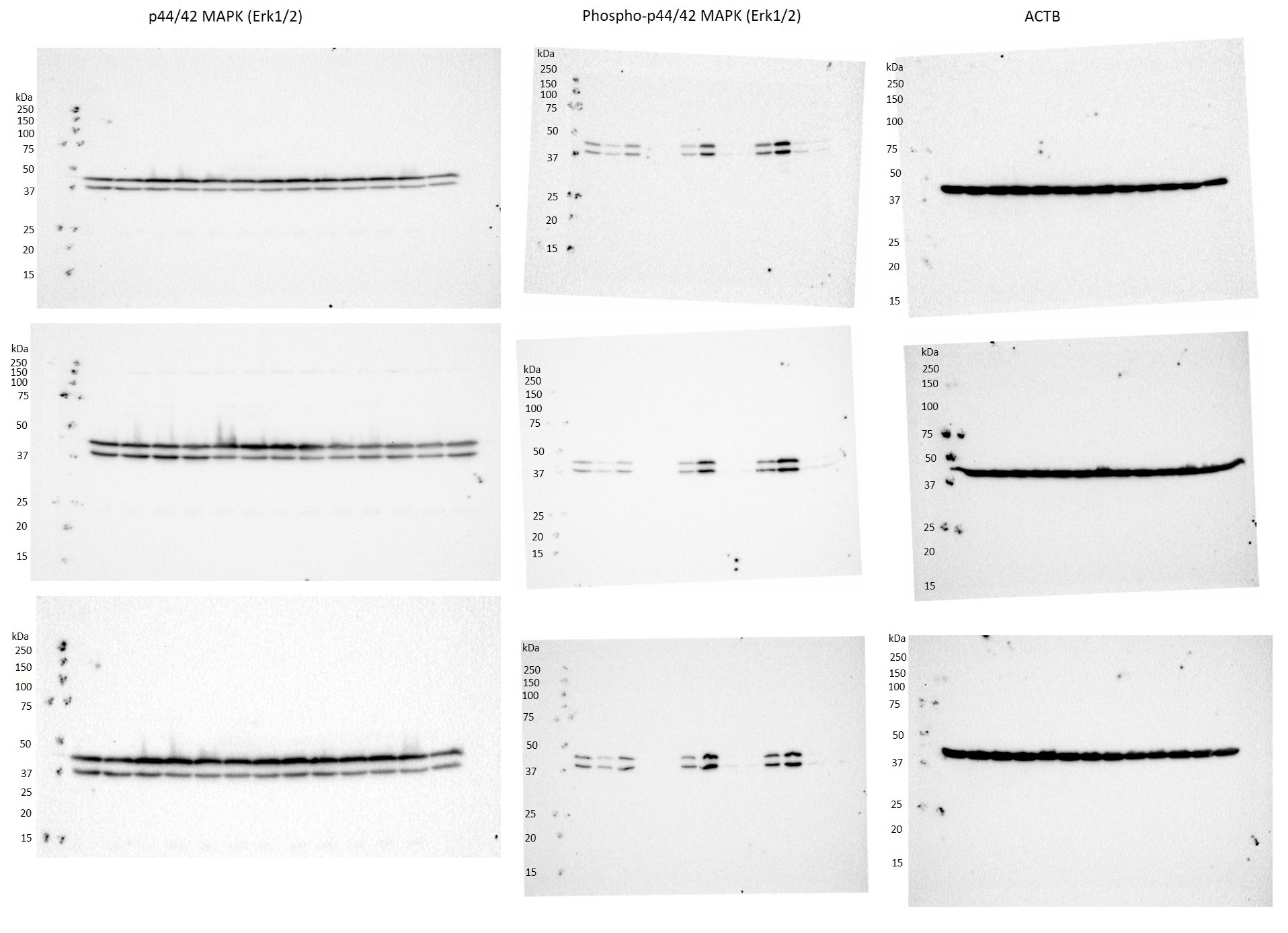

Supplement: Supplementary file 1 [file vetsci-09-00099-s001.zip › Figure S2.jpg]
